# Setting Health Targets Using Information from Probabilistic Projections: A Research Brief on an Application to Contraceptive Coverage

**DOI:** 10.1007/s11113-023-09766-2

**Published:** 2023-02-10

**Authors:** Ann Biddlecom, Elizabeth A. Sully, Vladimíra Kantorová, Mark C. Wheldon, Naomi Lince-Deroche, Taylor Riley

**Affiliations:** 1 Independent researcher, New York, NY USA; 2 Guttmacher Institute, New York, NY USA; 3 Population Division, Department of Economic and Social Affairs, United Nations, New York, NY USA; 4 Independent researcher, Athens, Greece; 5 Department of Epidemiology, University of Washington, Seattle, WA USA

**Keywords:** International health, Measurement, Evidence-based policy, Contraception

## Abstract

Broad and aspirational targets to meet health service needs are useful for advocacy, but setting measurable, time-defined targets for accelerated yet feasible progress is necessary for national monitoring and planning purposes. Information from probabilistic projections of health outcomes and service coverage can be used to set country-specific targets that reflect different starting points and rates of change. We show the utility of this approach in an application to contraceptive coverage in 131 low- and middle-income countries (LMICs) and the related cost and impact of different coverage scenarios. We use the sustainable development goal (SDG) indicator of the proportion of women who have their need for family planning satisfied with modern contraception. The results show that accelerated progress targets would collectively result in 83% of the need satisfied in 2030 for LMICs, which is 5% points higher than the projected level based on the current pace of progress. This translated into 41 million fewer women with an unmet need for modern methods and 14 million fewer unintended pregnancies. Annual direct costs would be $480 million more in 2030 to support contraceptive services compared with costs in 2030 based on the current pace of progress. As governments plan and budget for expanded health service coverage, information from probabilistic projections can guide them in setting measurable, ambitious yet realistic targets that are relevant to their particular contexts.

**Supplementary Information:**

The online version contains supplementary material available at 10.1007/s11113-023-09766-2.

## Introduction

All countries have committed to expanding essential health service coverage and improving people’s health and well-being as part of the United Nations Sustainable Development Goals (SDGs) and, in particular, achieving universal health coverage (United Nations, 2015). One challenge for monitoring progress towards these governmental commitments is to translate aspirational commitments to human well-being into measurable and achievable targets that take into account country-specific baselines and historical rates of progress. Put another way, idealized goals should be accompanied by realistic, precise, and actionable targets (Eyal & Sjöstrand, 2020). The SDG health-related targets include some that are precise but that set the same level to be achieved for all countries, regardless of their starting points (e.g., SDG Target 3.2 to reduce under-five and neonatal mortality in all countries to no more than 25 deaths and 12 deaths per 1000 births, respectively, by 2030). Other SDG targets are both imprecise and aspirational. For example, SDG Target 3.7 to ensure universal access to sexual and reproductive health care services by 2030 is imprecise because it lacks an empirically-formulated target and aspirational because it aims for universal access to services by 2030.

Each country is unique with regard to its population health needs and the capacity of the healthcare system to improve to meet those needs. Information about a country’s baseline level of health service coverage and its past experience in expanding coverage should inform expectations for improved performance in the near future. A common approach to setting targets is to fix the same level of coverage for all countries to reach within the same time period. This can lead to missed opportunities for progress for countries that are currently at or beyond the target level and yet still have gaps in coverage of essential health services (Kantorová et al., 2017). Conversely, the same fixed level of coverage ignores the wide variation in country-specific starting points and sets up unachievable targets for countries with low baseline levels of service coverage (Cahill et al., 2020; Choi et al., 2015; Kantorová et al., 2017). A related approach used, for example, in a landmark Lancet Commission on investments in global health draws on the experience of “best-performing countries” (i.e., countries that experienced historically rapid improvements in health outcomes and service coverage) as the basis for an accelerated progress scenario in which other countries scale-up service coverage (Jamison et al., 2013). This approach of “best-performing countries” has also been applied to set a common target for contraceptive coverage for all countries of at least 75% demand for family planning met with modern contraceptives (Fabic et al., 2015).

The increasing availability of probabilistic projections of country-specific, health-related indicators presents an opportunity to draw on information about uncertainty to assess change over time (Raftery, 2016). Information from country-specific probabilistic projections of health service needs and coverage is an under-utilized resource for setting measurable, realistic targets for accelerated progress in LMICs and to inform planning for the additional investments needed for and impact of reaching those targets.

To illustrate this approach, we use the example of contraceptive care, an essential sexual and reproductive health service. Governments and other key stakeholders have used aspirational targets or scenarios to close the gap *completely* between the proportion of people who want to avoid pregnancy and those using contraception (Johns Hopkins University et al., 2020; Sully et al., 2020; United Nations, 1999). While this may be useful for advocacy purposes and to call attention to the importance of this critical health- and human rights-related need, a target of 100% contraceptive use among people who want to prevent pregnancy is neither realistic nor appropriate in the context of individuals being able to make full, voluntary and informed decisions on whether to use contraception (Speizer et al., 2022). Time-defined targets that draw on country-specific information on the size of the gap in meeting potential demand for contraceptive methods and the pace of change in reducing that gap can be more informative for making accelerated progress.

## Application to Contraceptive Coverage

As part of monitoring the SDGs, countries track a demand satisfied indicator for contraception, which is defined as the proportion of women of reproductive age (aged 15–49 years) who have their need for family planning satisfied with modern methods (SDG indicator 3.7.1) (United Nations, 2017). We use probabilistic projections (unadjusted medians) of this indicator to 2030, based on the 2020 revision of family planning indicators (United Nations Department of Economic and Social Affairs, Population Division, 2020). Figures showing the full probabilistic projections, medians and distinct uncertainty intervals by country and indicator are available online (United Nations Department of Economic and Social Affairs, Population Division, 2020) and updated regularly. The data and software to generate the estimates and projections are publicly available (Wheldon et al., 2019).

The probabilistic projections are from a model that draws on more than 1300 survey data points; accounts for change in the population of women of reproductive age and in their marital status composition and sexual activity (Kantorová et al., 2020); and assesses uncertainty based on the extent and quality of underlying data. The model jointly produces estimates and projections of contraceptive prevalence (any, modern, traditional) and unmet need for family planning, and functions of these outcomes for other indicators like SDG indicator 3.7.1. We use 2019 estimates of the distribution of contraceptive methods used by women of reproductive age (Riley et al., 2020, Table MA4.3) and hold the method mix constant over time. The projections included in this analysis cover 131 low- and middle-income countries and represent women of reproductive age (15–49 years). Model-based outcomes are transformed into absolute numbers by multiplying the projected indicator value by the population of women of reproductive age in a country in the particular year. For simplicity, we present aggregated results for all 131 LMICs. Country-specific results are in Supplementary Appendix Table A1.

We use 2019 as the baseline and thus make no assumptions about the scale or duration of the COVID-19 pandemic’s impact on contraceptive service provision, use or need. Recent evidence from a scoping review provides no basis to make uniform assumptions about pandemic-related changes in contraceptive use or service provision (Polis et al., 2022). We use the country-specific change in the percent of demand satisfied by modern methods in 2019 and 2030 (the end point of the SDGs). A probability distribution of the magnitude of change between 2019 and 2030 is obtained for each country from the probabilistic projection model of contraceptive use. The “current progress” scenario uses the medians (50th percentile) of each country-specific probability distribution of change between 2019 and 2030. In short, there is a 50% chance that the change between 2019 and 2030 is at that level or higher in each country. The “current progress” scenario is what is likely to happen based on country-specific past trends.

For an “accelerated progress” scenario, the change should be from the upper end of the probability distribution for each country and yet not at an extreme point where there is a very small chance that the magnitude of change occurs. We chose the 90th percentile of the probability distribution in each country to reflect faster progress from 2019 than business as usual. At this level there is a one in ten chance that the change between 2019 and 2030 is at that level or higher in each country. One reason to avoid using information from the extreme ends of the probabilistic distribution (95th percentile and higher) is that countries with less data have larger uncertainty intervals. This overall approach accounts for uncertainty in the level of potential demand satisfied with modern methods in 2019 in each country because it uses probabilistic calculations throughout and is independent of the start and end values in 2019 and 2030.

We use the corresponding percent of need satisfied by modern methods and related family planning indicators (e.g., number of women using modern methods or with an unmet need for modern methods) that are jointly estimated under each scenario to estimate the impact on unintended pregnancy and direct costs for contraceptive services in 2030 for each country and aggregate the results for all LMICs. Impact is measured as annual number of unintended pregnancies, computed using age-adjusted contraceptive failure rates (Riley et al., 2020, Table MA7.1) and a pregnancy rate of 44.8% for women who use traditional contraceptive methods or who use no method at all. The latter rate is the median of the country-specific distribution of estimated pregnancy rates for this group, based on recent estimates of unintended pregnancy and abortion (Bearak et al., 2020, 2022). While it is possible that the pregnancy rate for these groups of women varies over time and place, we hold this rate constant in the absence of data that could inform an alternative approach.

Cost data are drawn from 2019 estimates (Riley et al., 2020, Table MA8.5) and updated with 2020 costs (data available here https://osf.io/qzrhw/). Personnel costs are inflated using country-specific inflation projections [i.e., Gross Domestic Product (GDP) Deflator data from the IMF] for 2021–2024 and the average of inflation in 2021–2024 for each year in the period 2025–2030. Drugs and supply costs are inflated using US inflation. We assume a constant annual inflation rate for the period 2021–2030, equivalent to the average for the period 2015–2019. Contraceptive commodity costs are not inflated for the period 2020–2030. This is a conservative approach as observed prices for commodities relevant to this analysis, supplied by the United Nations Population Fund (UNFPA) and other suppliers, have decreased during the period 2015–2019. Finally, the total average cost per modern contraceptive method per year is the sum of the cost components noted above: personnel, drugs and supplies, and the commodity itself.

Table 1 shows that if contraceptive coverage continues apace under the “current progress” scenario in LMICs, then, in the aggregate, 78% of women of reproductive age who want to avoid pregnancy would be using modern methods in 2030. This leaves 218 million women who want to avoid pregnancy but are not using a modern method and 97 million unintended pregnancies. Direct costs in 2030 for 783 million modern method users in LMICs would be nearly $4 billion dollars. For comparison, direct costs in 2019 were estimated to be $3.5 billion dollars (Sully et al., 2020).

**Table 1:** Progress, impact and cost in 2030 under different targets for meeting potential demand for modern contraceptive methods in 131 low- and middle-income countries

Projections	Current progress	Accelerated progress	Difference (absolute), accelerated—current
% of demand satisfied by modern methods	78%	83%	5%
No. of women using modern methods (000 s)	783,000	876,000	93,000
No. of women with an unmet need for modern methods (000 s)	218,000	177,000	41,000
No. of unintended pregnancies (000 s)	97,000	83,100	13,900
Annual total direct costs ($) (000 s)	$3,990,000	$4,470,000	$480,000

Accelerated progress across countries would result in 83% of potential demand satisfied with modern methods in LMICs in 2030 (an increase of 5% points), or 93 million more modern method users in 2030 than would be expected under the current pace of progress. There would also be 41 million fewer women with an unmet need for modern methods and 14 million fewer unintended pregnancies in 2030. Annual direct costs for contraceptive services would be $480 million more in 2030 under accelerated targets compared with the current pace of progress. These projected cost estimates do not account for cost savings attributable to additional unintended pregnancies averted and their consequent pregnancy-related care costs (see, for example, Jamison et al., 2013; Lince-Deroche et al., 2020; Sully et al., 2020).

Setting universal or aspirational targets or those based on successful experiences of particular countries may motivate political commitment and resource allocation, but this practice is not useful for determining realistic investments and national budgets, adequate planning, and accountability mechanisms to support country-specific progress. For example, even under an accelerated progress in this application, 62 LMICs have coverage under 75% in 2030 on this SDG indicator (Supplementary Appendix Table A1), below the average for all LMICs under the current progress scenario as well as a proposed target based, in part, on “best-performing countries” (Fabic et al., 2015). The benefit of using information from probabilistic projections is that it is a data-informed, country-specific customization of what is an ambitious target.

Over this next decade, as governments renew efforts to expand coverage of essential health services, information from probabilistic projections of health-related outcomes and services can be useful to chart the unique course forward in each country. This approach also aligns with an increasing focus in international initiatives on country-led commitments and monitoring (e.g., the FP2030 global partnership to advance rights-based family planning). We showed aggregated results from country-specific projections for two scenarios, but probabilistic information can also be used to construct ranges under different levels of acceleration (e.g., gradual acceleration at the 60th percentile) as well as other types of scenarios (e.g., delayed acceleration given an external shock, such as an epidemic or civil strife). This research application of the often-overlooked value of information about uncertainty highlights its policy-relevant utility, especially as probabilistic projections of health outcomes and services become increasingly available in countries worldwide.

## Supplementary Information

Below is the link to the electronic supplementary material.

Supplementary file1 (XLSX 45 KB)

## Data Availability

The software to generate the estimates and projections of family planning indicators, including the MCMC trajectories and summary quantiles, is available from a public GitHub repository. A suggested citation is: Wheldon et al. (2019). Detailed cost data for 2020 are available here https://osf.io/qzrhw/. Contraceptive method mix data are available here https://osf.io/m85s9/. Stata code and data files to generate the research brief results and supplementary table are available here https://osf.io/mnarw/.

## References

[CR1] Bearak JM, Popinchalk A, Ganatra B, Moller A-B, Tunçalp Ö, Beavin C, Kwok L, Alkema L (2020). Unintended pregnancy and abortion by income, region, and the legal status of abortion: Estimates from a comprehensive model for 1990–2019. The Lancet Global Health.

[CR2] Bearak JM, Popinchalk A, Beavin C, Ganatra B, Moller A-B, Tunçalp Ö, Alkema L (2022). Country-specific estimates of unintended pregnancy and abortion incidence: A global comparative analysis of levels in 2015–2019. BMJ Global Health.

[CR3] Cahill N, Weinberger M, Alkema L (2020). What increase in modern contraceptive use is needed in FP2020 countries to reach 75% demand satisfied by 2030? An assessment using the accelerated transition method and family planning estimation model. Gates Open Research.

[CR4] Choi Y, Fabic MS, Hounton S, Koroma D (2015). Meeting demand for family planning within a generation: Prospects and implications at country level. Global Health Action.

[CR5] Eyal N, Sjöstrand M (2020). On knowingly setting unrealistic goals in public health. American Journal of Public Health.

[CR6] Fabic MS, Choi Y, Bongaarts J, Darroch JE, Ross JA, Stover J, Tsui AO, Upadhyay J, Starbird E (2015). Meeting demand for family planning within a generation: The post-2015 agenda. The Lancet.

[CR7] Jamison DT, Summers LH, Alleyne G, Arrow KJ, Berkley S, Binagwaho A, Bustreo F (2013). Global health 2035: A world converging within a generation. The Lancet.

[CR8] Johns Hopkins University, Avenir Health, Victoria University, Institute of Health, Metrics and Evaluation at the University of Washington, & UNFPA. (2020). *Costing the three transformative results*. UNFPA.

[CR9] Kantorová V, Rou J, Biddlecom A, Alkema L (2017). Setting ambitious yet achievable targets using probabilistic projections: Meeting demand for family planning. Studies in Family Planning.

[CR10] Kantorová V, Wheldon MC, Ueffing P, Dasgupta ANZ (2020). Estimating progress towards meeting women’s contraceptive needs in 185 countries: A Bayesian hierarchical modelling study. PLoS Medicine.

[CR11] Lince-Deroche N, Sully EA, Firestein L, Riley T (2020). Budgeting for comprehensive sexual and reproductive health and rights under universal health coverage. Sexual and Reproductive Health Matters.

[CR12] Polis CB, Biddlecom A, Singh S, Ushie BA, Rosman L, Saad A (2022). Impacts of COVID-19 on contraceptive and abortion services in low- and middle-income countries: A scoping review. Sexual and Reproductive Health Matters.

[CR13] Raftery AE (2016). Use and communication of probabilistic forecasts. Statistical Analysis and Data Mining: THe ASA Data Science Journal.

[CR14] Riley T, Sully EA, Lince-Deroche N, Firestein L, Murro R, Biddlecom A, Darroch JE (2020). Adding it up: Investing in sexual and reproductive health 2019—methodology report. Guttmacher Institute, New York.

[CR15] Speizer IS, Bremner J, Farid S, FP2020 Performance, Monitoring and Evidence Working Group (2022). Language and measurement of contraceptive need and making these indicators more meaningful for measuring fertility intentions of women and girls. Global Health Science and Practice.

[CR16] Sully, E., Biddlecom, A., Darroch, J. E., Riley, T., Lince-Deroche, N., Firestein, L., & Murro, R. (2020). *Adding it up: Investing in sexual and reproductive health 2019*. Guttmacher Institute, New York. 10.1363/2020.31593.

[CR17] United Nations. (1999). *Key actions for the further implementation of the programme of action of the international conference on population and development*. (Resolution Adopted by the General Assembly on 2 July 1999 A/RES/S-21/2). United Nations General Assembly.

[CR18] United Nations. (2015). Transforming our world: The 2030 agenda for sustainable development. (Resolution Adopted by the General Assembly on 25 September 2015 A/RES/70/1). United Nations General Assembly. Retrieved from http://undocs.org/en/A/RES/70/1.

[CR19] United Nations. (2017). *Work of the statistical commission pertaining to the 2030 agenda for sustainable development* (Resolution Adopted by the General Assembly on 6 July 2017 A/RES/71/313). United Nations General Assembly. Retrieved from http://undocs.org/en/A/RES/71/313.

[CR20] United Nations, Department of Economic and Social Affairs, Population Division. (2020). *Estimates and projections of family planning indicators 2020*. United Nations. Retrieved from https://www.un.org/en/development/desa/population/publications/pdf/family/Figure_Model-based_estimates_Countries_2020.pdf.

[CR21] Wheldon, M. C., New, J. R., Cahill, N., Gu, G., Tait, A., Hertog, S., & Alkema, L. (2019). *FPEMglobal* (1.0.0) [R]. GitHub. Retrieved from https://github.com/FPcounts/FPEMglobal/tree/v1.0.0.

